# Determination of Seroprevalence and Risk Factors of Crimean–Congo Haemorrhagic Fever (CCHF) in the Endemic Region in Turkey: A Population-Based Cross-Sectional Study

**DOI:** 10.1155/2021/9945089

**Published:** 2021-05-17

**Authors:** Rıza Çıtıl, Mücahit Eğri, Yalçın Önder, Fazilet Duygu, Yunus Emre Bulut, Özkan Yaşayancan, Nagehan Yıldız Çeltek, Şafak Şahin

**Affiliations:** ^1^Tokat Gaziosmanpaşa University, Faculty of Medicine, Department of Public Health, Tokat, Turkey; ^2^Goethe University Frankfurt, Department of Internal Medicine, Infectious Diseases, Frankfurt, Germany; ^3^Public Health Specialist, Ankara Public Health Directorate, Ankara, Turkey; ^4^Public Health Specialist, Samsun Public Health Directorate, Samsun, Turkey; ^5^Tokat Gaziosmanpaşa University, Faculty of Medicine, Department of Family Medicine, Tokat, Turkey; ^6^Tokat Gaziosmanpaşa University, Faculty of Medicine, Department of Internal Medicine, Tokat, Turkey

## Abstract

**Objectives:**

Turkey is one of the countries that has the most cases of CCHF in recent years among the endemic countries. The disease also poses an important health threat with high mortality rate. The aim of the study was to determine the seroprevalence and risk factors of CCHF in adults aged ≥20 years in Tokat in the endemic region, Turkey.

**Methods:**

In this population-based cross-sectional study, a total of 85 Family Medicine Units (FMUs), from over 170 in Tokat, were randomly selected using 50% sampling. The sample size was determined among the subjects aged ≥20 who registered with the FMUs, due to gender, age group, and the urban/rural population size of Tokat using the stratified cluster sampling method. Subjects were invited to the FMUs. A questionnaire was performed face to face. The blood samples were taken, and anti-CCHFV IgG antibodies were measured with ELISA method.

**Results:**

1272 (54.9%) out of 2319 participants were female, and the mean age was 47.3 ± 15.3. Anti-CCHFV IgG seropositivity was 5.6% (*n* = 130). Seropositivity rates in terms of adjusted odds ratios (AOR) were higher 2.53 times (95% CI: 1.57–4.08; *p*=0.001) in males; 4.05 (95% CI: 2.14–7.65; *p* < 0.001) in age group ≥65; 0.33 (95% CI: 0.14–0.76; *p* < 0.001) in graduates of high school and above; 0.71 (95%CI: 0.33–1.52; *p* < 0.001) in ones with good income; 1.84 (95%CI: 1.18–2.86; *p* < 0.001) in farmers; 1.64 (95% CI: 1.04–2.27; *p* < 0.001) in people dealing with animal husbandry; and 1.02 (95% CI: 1.03–2.29; *p* < 0.001) in those with history of tick contact.

**Conclusions:**

CCHF seroprevalence is still a common public health problem in Tokat, Turkey. Male gender, advanced age group, low-educated, low-income, farmers, animal husbandry, and history of tick contact were found to be risk factors for CCHF. The importance of this kind of community-based studies to identify the seroprevalence in regional and national level increases even more.

## 1. Introduction

Disease transmitted by ticks is Rocky Mountain spotted fever (*Rickettsia*), Boutonneuse (*Rickettsia*), Q-fever (*Rickettsia*), Endemic typhus (*Rickettsia*), Tularemia (*Rickettsia*), Lyme (*Borrelia*), Relapsing fever (*Borrelia*), Colorado tick-fever (viruses), Arboviruses (viruses), and Crimean–Congo Hemorrhagic Fever (CCHF). CCHF is a tick-borne viral, zoonotic, infectious disease caused by Nairoviruses, transmitted by *Ixodidae* and *Argasidae* family ticks, posing a significant health threat as it causes fatal haemorrhagic syndrome in humans [1]. CCHF is currently endemic in about 50 countries in Europe, Africa, and Asia, with seroprevalence ranging within 0.1–14.4% in the general population, 16.5–30.3% in at-risk professionals, and 18.5–85% in patient-related populations [1]. The fatality of the disease changes from region to region in the world; however, it is reported as 5–30% [2].

Ticks are now accepted as both the vector and the reservoir for the CCHF virus. It is declared that the distribution and reservoirs of the known tick types get common additional globalisation and constantly increasing international animal transportation cause the spread of pathogens and vectors globally [3]. It is stated that more than 30 tick types are capable of living in Turkey's geographical conditions [4]. The species of *Hyalomma* genus ticks are the main CCHF vectors in Turkey. It is known that many complex interrelated factors contribute to the emergence and spread of the CCHF, such as environmental and climate changes, and the uncontrolled movement of wild birds and animals [5]. The main modes of transmission of CCHF are tick contact, viraemic animals, and infected body fluids/blood. The most common symptoms are high fever, headache, fatigue, myalgia, and nausea. Later on, haemorrhagic symptoms could show [6]. It is reported that anti-Crimean-Congo haemorrhagic fever virus (anti-CCHFV) immunoglobulin M (IgM) antibodies could be detected in 6–40 days following the entrance of virus to the body and anti-CCHFV immunoglobulin G (IgG) antibodies could be detected between 10^th^ day to 6^th^ year following the infection [7]. Though the local vaccine studies which will play an important role in protection against the disease are ongoing, there has not been a development of a specific antiviral to be used in treatment or a vaccine with world-wide recognised efficacy.

**Figure 1:**
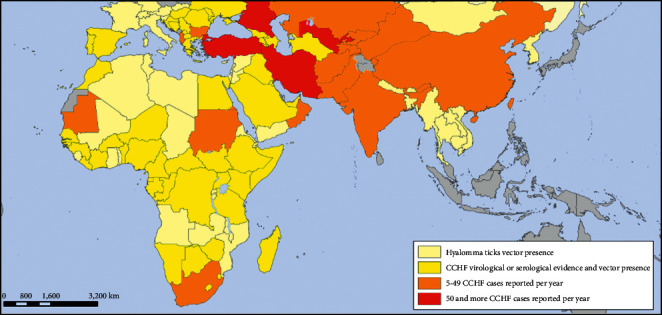
World-wide map of CCHF distribution [2].

The first CCHF case in Turkey was reported in 2002 in Tokat province [8]. Turkey is one of the endemic countries that has the most cases of CCHF in recent years [9, 10]. The disease is endemic in cities in the region known as “Kelkit Basin” (Tokat, Artvin, Bayburt, Erzincan, Erzurum Gümüşhane, Amasya, Çankırı, Çorum, Kastamonu, Sivas, Yozgat) [4, 5, 8] (Figure 1).

Mortality rates change in accordance with the geographical region (4–20%) and with this high mortality rate; CCHF is an important public health problem in Turkey [11, 12]. All CCHF cases are monitored by a strong surveillance system by the Ministry of Health. In Turkey, between the years 2002 and 2018, 11.041 cases of CCHF have been reported and the case-mortality rate was 4.8% [13]. Although it varies from region to region and in the groups studied, the seroprevalence of CCHF in endemic areas is determined to be 0.7% to 19.4% [10, 14–16]. Determining the CCHF seroprevalence is important to understand the epidemiology of the disease better and taking the necessary precautions on time [10]. Regarding CCHF, in literature, apart from the specific groups and few seroprevalence studies in a limited participation, there are not enough studies representing healthy individuals especially in Tokat where the first case in Turkey was detected. In this study, it was aimed at determining seroprevalence and related risk factors of CCHF in adults aged ≥20 years in Tokat in the endemic region, Turkey.

## 2. Methods

The population-based cross-sectional study was carried out in the city of Tokat, which is in the middle Black Sea region of Turkey. In this study, the population of Tokat province ≥20 years consists of 412.653 individuals. In calculating the sample size, the expected prevalence of the disease to be investigated was found to be 50%, the deviation was taken as 0.05, and the design effect was taken as 2 at 97% confidence level and the population targeted to be reached with the Epi Info version 7 program was found to be 2635.

The sample selection was made by multi-layer proportional cluster sampling method considering the size of the urban and rural settlements of the provincial centres and districts in the Tokat provincial population pyramid, gender, and age groups. A total of 85 Family Medicine Units (FMU) from over 170 in Tokat were randomly selected using a 50% sampling. Each FMU was considered as a cluster. By using the quota sampling method in the intracluster sample, the number of individuals required to fall to the determined gender and age groups is provided to work. The gender and age groups whose numbers were set for each cluster were randomly selected by systematic sampling method after ranking by Family Medicine Information System. The participants included in the study were invited to the FMUs. All participants signed a voluntary consent form. Those who reported cognitive impairment that would prevent the questionnaire forms from understanding or giving clear answers and those who declared that they were pregnant were excluded from the study.

For the data collection, a questionnaire about the sociodemographic characteristics and CCHF-related risk factors of the participants was completed by face-to-face interviews by physicians in study group and blood samples were taken from the participants for laboratory tests. The sera obtained by centrifuging the samples were stored at +4°C and then transferred to a −70°C unit at the end of the day until further analysis could be performed. Anti-CCHFV IgG were studied by serum in the Public Health Institution of Turkey Microbiology Reference Laboratory. Specific IgG antibody level against CCHFV was measured in serum with the Enzyme-Linked Immuno Sorbent Assay (ELISA) method which is a method that can be used in serosurveillance studies. 92% (2428) of the sample calculated in the study was reached. As a result, the data of 2319 (88%) participants were included in the statistical analysis due to insufficient blood samples or inability to analyze serum samples from 109 participants. The dependent variables were anti-CCHFV IgG serology and evaluated in two categories as seropositive and seronegative. The independent variables were some descriptive characteristics and CCHF-related risk factors.

### 2.1. Statistical Analysis

The data analysis was performed using SPSS 22.0. Categorical variables were summarized by number, percentage, mean ± standard deviation (min-max), and 95% confidence interval, compared with Pearson Chi-square and Fisher's exact tests. Logistic regression analysis was used for the descriptive and CCHF-related risk factors for anti-CCHFV IgG seropositivity. The values of *p* < 0.05 were considered as statistically significant.

### 2.2. Ethical Approval

The study was approved by the Ethical Committee of Tokat Gaziosmanpaşa University Faculty of Medicine (approval number: 14-KAEK-142). A written informed consent was obtained from each participant.

## 3. Results

### 3.1. Sociodemographic Characteristics

54.9% (1272) of 2319 participants were female, and the mean age was 47.3 ± 15.3 (20–87 ages). 69.2% of the participants lived in the districts, 58.3% of them were graduates of primary and secondary school, 47.9% of them were housewives, 86.8% were married, and 51.3% had poor level of family income (Table 1).

### 3.2. CCHF-Related Risk Factors

According to some risk factors for CCHF of the participants, 41.6% of them lived in rural areas, 15.1% were farmers, 33.1% were involved in animal husbandry, and 16% had tick-contact history. 1% of the participants had hospitalization history with suspected CCHF, 7% of those had a relative with history of CCHF treatment, and in the area where 15.5% of participants lived there were people diagnosed with CCHF (Table 2).

### 3.3. Seroprevalance of CCHF

Anti-CCHFV IgG seropositivity was 5.6% (*n* = 130) (4.2% in females, *n* = 53, 7.4% in males, *n* = 77). The mean age of seropositives (57.7 ± 15.2) was higher than those of seronegatives (46.7 ± 15.1) (*p* < 0.001). The highest seropositivity rate was 10.9% in the 60-age group (14.2% in males, 8.1% in females). A statistically significant difference was found for anti-CCHFV IgG seropositivity by age groups in both genders, and the seropositivity increased with advancing age (*p*=0.001 in females; *p* < 0.001 in males) (Table 3).

In the logistic regression analysis of the descriptive characteristics of the participants in terms of anti-CCHFV IgG seropositivity (Table 4), gender (*p*=0.001), age group (*p* < 0.001), education level (*p* < 0.001), marital status (*p*=0.014), and income level (*p* < 0.001) were found to have significant effects on seropositivity. The rates of seropositivity in terms of adjusted odds ratios (AOR) were higher 2.53 times (95% confidence interval (CI): 1.57–4.08) in males; 4.05 (95% CI: 2.14–7.65) in age group ≥65; 0.33 (95% CI: 0.14–0.76) for those whose education level was high school and above; and 0.71 (95%CI: 0.33–1.52) in those with good income.

In the logistic regression analysis of some CCHF-related risk factors in terms of anti-CCHFV IgG seropositivity of the participants, it was identified that farming, dealing with animal husbandry, history of tick contact, and history of hospitalization with suspected CCHF and being anyone diagnosed with CCHF in the place of residence significantly affected the seropositivity. Seropositivity rates in terms of AOR were higher 1.84 times in farmers (AOR: 1.84; 95%CI: 1.18–2.86; *p* < 0.001); 1.64 in people dealing with animal husbandry (AOR: 1.64; 95%CI: 1.04–2.27; *p* < 0.001); 1.02 in those with a history of tick contact (AOR: 1.02; 95%CI: 1.03–2.29; *p* < 0.001); 6.65 in those hospitalized with suspected CCHF (AOR: 6.65; 95%CI: 2.70–16.43; *p* < 0.001); and 1.66 in being anyone diagnosed with CCHF in place of residence (AOR: 1.66; 95%CI: 1.08–2.56; *p* < 0.001) (Table 5).

## 4. Discussion

Although the morbidity and mortality of CCHF in Tokat province, where CCHF is endemic, have been reported to be decreasing compared with previous years [13], seroprevalence of the disease is still common in our region. According to the results of our study, the population in the at-risk group that makes a living from agriculture and animal husbandry is considerable. Although the proportion of those living in rural areas is less, those in the city centre often have a history of visit to rural areas. A history of tick contact was detected in 16% of the participants. The main reasons for the high rate are as follows; it is an endemic region, the participants live in rural areas, and they are risky individuals for CCHF as they deal with farming and animal husbandry. Our study reveals that a significant number of participants, either themselves or those around them, have had serious experiences in CCHF, such as history of hospitalization with suspected CCHF (1%), a family member that had been treated for CCHF (7%), and someone with diagnosed of CCHF in their circle (15.5%). Similar to our results, in a study comprising healthy people in Turkey, it was determined that 14.4% of the subjects had a history of tick bites [15]. In a seroprevalence study conducted in Turkey, it was found that 11% of the participants had a relative infected with the virus [16].

Anti-CCHFV IgG seropositivity was found as 5.6% (*n* = 130) in our study. In a meta-analysis in which studies on CCHF seroprevalence in different regions of the world were examined, it was reported that this rate varied between 0.1% and 14.4% in healthy individuals and it is the highest seroprevalence rate in Turkey [1]. In a systematic review conducted in World Health Organization (WHO) European Region, CCHF seroprevalence was found lowest in Spain (0%) and highest in Turkey (19.6%). Although there is low endemicity in Southern and Western European countries like Greece and Spain, the highest seroprevalence was detected in Central and Eastern Europe. In some countries, a neighbor of Turkey, if the anti-CCHFV IgG seropositivity is evaluated, the northeast of Turkey, especially the areas surrounding the Black Sea, are described as highly endemic for CCHFV. The highest rate of CCHFV seroprevalence was determined in Turkey, the Russian Federation, and Kazakhstan. Although Balkan countries are also considered endemic for CCHF, the seroprevalence was reported to be lower than in Turkey [17]. Anti-CCHFV IgG seropositivity was identified as 2.8% in healthy adults in a study with similar sampling method used in Bulgaria and 3.7% in Christova et al.'s study [18, 19]. The seroprevalence in Greece was reported to be between 2.2% and 4.2% [1]. The movement of livestock and ticks plays an important role in the disease transmission. The study conducted in Iran showed a relatively high frequency of the disease in individuals at risk (14.8%). It was reported that most of the cases are from the southeastern regions of Iran and infected livestock imported from the eastern provinces is one of the most common causes of the prevalence of the disease [7].

In a systematic review that examined the seroprevalence studies in Turkey, CCHF seroprevalence was reported as 0.5%–19.6% [17]. In a study conducted in Turkey's seven provinces representing seven geographical regions and based on layered sample in accordance with the gender and age of adults, in 1066 venous blood samples representing 48.5 million adult anti-CCHFV IgG antibodies were detected with ELISA method, and CCHF seroprevalence was identified as 2.3%. In the same study, seroprevalence rates in Adana, Aydın, Erzurum, Gaziantep, İstanbul, Samsun, and Yozgat provinces were 0.7%–7.5% [10]. In Trabzon, which is endemic region in terms of CCHF, the seroprevalence was 13.6% in people living in the same environment with CCHF cases [20]. In the study performed in Van, CCHF seroprevalence was 14.4% in healthy individuals [15]. In another study performed in three endemic regions in Aydın, the highest rate (19.6%) was found among the CCHF seroprevalence studies in Turkey [16]. The seroprevalence rate found in our study is lower than the results of these studies conducted in Trabzon, Van, and Aydın provinces. With regard to the results of retrospective studies in regions where CCHF is endemic in Turkey, it has been reported that CCHF incidence and mortality rates decreased over the years [11, 21, 22]. When the results in our study area are compared with the results of other areas in terms of endemicity, it is thought that the main reasons for the differences are that the participants are healthy individuals rather than high-risk occupational groups and the study is a community-based study that adequately represents the general population in our province. In addition, as stated in studies conducted in high-risk endemic regions, the increased experience and awareness of both individuals and healthcare professionals in those regions on tick contact and CCHF may have an effect on lower incidence and mortality rates compared to the past [21].

In our study, anti-CCHFV IgG seropositivity was 4.2% in females and 7.4% in males. The mean age was significantly higher in seropositive. Seropositivity was highest in the age group of ≥60 (10.9%) and increased with advancing age. Seropositivity was statistically significantly higher in males and age group ≥65 years. In our study, the male gender was found to be a risk factor for CCHF similar to the study representing the seven geographical regions of Turkey [10]. The reason for this is that it is thought that men spend more time in jobs such as farming and animal husbandry. These activities are mostly performed by men in the countries the most at risk, as in the Middle East. This gender difference varies between countries depending on the participation of women in agricultural work [2, 16]. In opposition to that, in the study conducted in Greece, it is identified that the female gender is risky in terms of CCHF [23]. Also, it is reported that gender does not significantly affect anti-CCHFV IgG seropositivity according to the studies performed in Bulgaria [18], and in Erzurum [4], Van [15], Erzincan [11], and Trabzon [20] in Turkey. Similar to our study, many studies in the literature have shown that increased age was an important risk factor which may be a result of the increased possibilities for transmission [4, 10, 20, 23–25]. The studies have shown that age has no significant effect on seroprevalence contrary to our study [8, 18]. In the study performed in Aydın, the seroprevalence was found to be dramatically higher in the <34 age group, contrary to both our studies and also the ones in the literature [16].

In our study, low education and low-income levels were found to be risk factors for anti-CCHFV IgG seropositivity. Seropositivity was lower in graduated from high school and in those with good income significantly (*p* < 0.001). In a study, similar to our results, low education level was found to be a risk factor in terms of seropositivity [10]. The main reason for this is that farming and animal husbandry are generally more common among the low-educated and low-income population. In our study, farming, animal husbandry, history of tick contact, and hospitalization with suspected CCHF and being an individual with a CCHF diagnosis in their place of residence significantly affected anti-CCHFV IgG seropositivity (*p* < 0.001). Seropositivity was higher 1.84 times in farmers, 1.64 in animal husbandry, 1.02 in those with a history of tick contact, 6.65 in those hospitalized with suspected CCHF, and 1.66 in individuals with a diagnosis of CCHF where they lived. It has been reported that the most common risk factors for CCHF seropositivity are animal contact, animal husbandry or farming, history of tick contact, being a housewife, and exposure to secretions risky for CCHF [1]. In the study conducted in an endemic region in Turkey, tick exposure (OR: 9.03; 95%CI: 1.96–41.47; *p*=0.005) was found to be an independent indicator for CCHF [26].

In the literature, it has been stated that living in rural areas is a risk factor in terms of exposure to ticks and CCHF [1, 11, 19, 20, 27]. In our study, 41.6% of the participants lived in rural areas; there was not a difference in seropositivity between rural (6.2%) and urban (5.2%) areas (*p*=0.28). Similar to our study results, it was found in a study conducted in Van that locality did not have a significant effect on anti-CCHFV IgG seropositivity [15]. In the study conducted in Erzurum, which is endemic in terms of CCHF, the frequency of anti-CCHFV antibodies was found to be significantly higher in those living in rural areas [4]. In a study conducted in Erzincan, which is endemic for CCHF in Turkey, between the years 2011–2017, it was identified that the vast majority of CCHF patients (94.2%) were living in rural areas [11]. Unlike our study, it is thought that the prevalence of CCHF was found to be significantly higher in rural areas, since this study was conducted with CCHF patients. However, our study is a seroepidemiological prevalence study in which healthy individuals were included. Unlike our study results, Yağcı-Çağlayık et al. found that CCHF seroprevalence was 4.1% in rural areas and 1.8% in urban areas; these rates were found to be higher in rural areas compared to urban areas in Aydın (4.1–0%), Istanbul (5.0–1.7%), Samsun (2.6–0%), and Yozgat (16.7–4.8%) [10]. In addition to that in Erzincan anti-CCHFV IgG positivity was found to be significantly higher in individuals who were engaged in animal husbandry in rural areas and had a history of tick exposure compared to individuals who were not exposed to ticks in the urban areas (*p* < 0.05) [14]. In the study, in which the medical data of 1258 patients who were admitted to the university hospital emergency service with tick contact between 2012 and 2018 in Tokat were analyzed retrospectively, 45.8% of the applicants were from the provincial centre, 47.4% from the district centre, and 6.8% from the town or village. This result has been interpreted as individuals dealing with agriculture and animal husbandry prefer to reside in provinces and districts as for more advanced opportunities [22]. It has been reported in the literature that CCHF seroprevalence is higher in urban areas, as livestock markets are generally located near large cities [28]. In a meta-analysis, it was reported that CCHF seroprevalence in individuals in the risk group world-wide is 7.5 times higher than in other healthy people in the community [1]. In studies conducted in different regions of Turkey, it has been shown that occupational exposure has significant impact on the incidence rate of the disease [4, 10]. The significance of the farming risk factor for CCHF has decreased over the years, as the CCHF seropositivity rates have been decreasing in recent years in farmers world-wide [1]. In the study conducted in Erzurum, the seroprevalence of anti-CCHFV antibodies was significantly higher in those dealing with animal husbandry [4].

Many studies have shown that the history of tick bite is an important risk factor for CCHF [1, 10, 14, 16, 29]. In the study conducted in Aydın, 41.1% of the participants had tick contact history and a relationship was found between IgG positivity and tick contact significantly [16]. In the study conducted by Karakeçili et al. , 77.2% of the patients were engaged in farming or animal husbandry and the bare-handed tick contact was high (52.9%) [11]. Tick bites and dealing with animal husbandry are well-known risk factors for CCHF. These risk factors were reported in the majority of CCHF cases in Turkey [29]. On the other hand, there are studies that the history of tick bite did not significantly affect anti-CCHFV IgG seropositivity [4, 15]. In the study conducted in Trabzon, animal husbandry (OR: 1.84, 95% CI: 1.09–3.11), contact with ticks (OR: 3.45, 95% CI: 1.87–6.46), and removing ticks from animals with bare-hands (OR: 2.48, 95% CI: 1.48–4.18) were associated with an increase in IgG seropositivity [20]. It is stated that the “One Health” concept is a suitable approach in the development of strategies to fight against zoonotic infectious diseases [30]. Studies emphasized the importance of this approach for tick-borne diseases such as CCHF, and all relevant professionals are called to join their efforts in combating these diseases [31]. Especially among healthcare professionals working in high-risk areas, activities that increase awareness of CCHF prevention, early diagnosis, and required treatment should be continued [32]. It is stated that more studies, especially multi-centered seroprevalence studies with broader participation, are needed to determine the current state of the CCHF epidemiology and to establish standard guidelines for taking necessary initiatives in endemic regions [18].

### 4.1. Limitations and Strengths

This study has some limitations. First of all, since it is a cross-sectional study, it can only be generalized to the province of Tokat where the study was conducted. Second, since only individuals ≥20 years old were included in our study, it is not possible to obtain information about CCHF seroprevalence in younger age group. Finally, it should not be disregarded that the actual prevalence could be higher than the identified as there may be people who have been exposed to the CCHFV and whose anti-CCHFV IgG antibodies have not been detected at a diagnostic level as a consequence of antibody level decrease following years of the infection. Despite all these limitations, in terms of the identification of CCHF seroprevalence and relevant risk factors at utmost accuracy, one of the strengths of the study that it was conducted not among the patients referred to healthcare centres or occupationally risky group but among a seemingly healthy population included in a layered sample representing the community in general based on gender, age group, and urban or rural residents in Tokat province located in Kelkit Basin where it is endemic for CCHF in Turkey. Besides, the participation rate to the study is quite sufficient for such a field study.

## 5. Conclusion

In conclusion, in this population-based epidemiological study conducted in adults aged ≥20 years in Tokat in the endemic region, it was found that CCHF seroprevalence is common. Anti-CCHFV IgG seropositivity was significantly high in males, advanced age group, low-educated, low-income, farming and/or animal husbandry, history of tick contact, hospitalization with suspected CCHF, and having individuals with diagnosed CCHF in the place of residence. The results of our study revealed the necessity of increasing measures for public health. It should be kept in mind that CCHF control is not only the duty of healthcare professionals, but multisectoral cooperation is required within the framework of the “One Health” concept. As CCHF is a common and important public health issue, such studies to be realized to identify the seroprevalence in regional and national level with community-based approach gain more importance.

## Figures and Tables

**Table 1:** Distribution of the participants according to some descriptive characteristics.

Variables	Categories	*n* (%)
Gender	Female	1272 (54.9)
	Male	1047 (45.1)
Age groups	20–39 years	925 (39.9)
	40–64 years	1025 (44.2)
	≥65 years	369 (15.9)
Residential area	City center	714 (30.8)
	Districts	1605 (69.2)
Education level	Illiterate-literate	412 (17.8)
	Primary school-secondary school	1353 (58.3)
	High school-university	554 (23.9)
Occupation	Housewife	1110 (47.9)
	Farmer	351 (15.1)
	Worker	224 (9.7)
	Retired	203 (8.8)
	Other (officer, tradesman, others)	431 (18.6)
Marital status	Married	2012 (86.8)
	Single	142 (6.1)
	Divorced	165 (7.1)
Family income status	Bad	1189 (51.3)
	Medium	718 (31)
	Good	412 (17.8)
Chronic disease	No	1087 (46.9)
	Yes	1232 (53.1)
Smoking	No	1451 (62.6)
	Yes	868 (37.4)
Alcohol use	No	2104 (90.7)
	Yes	215 (9.3)
Obesity (body mass index ≥30 kg/m^2^)	No	1416 (61.1)
	Yes	903 (38.9)
Total		2319 (100.0)

**Table 2:** Distribution of participants according to their characteristics related to some risk factors for CCHF.

Variables	Categories	*n* (%)
Location	Urban	1354 (58.4)
	Rural	965 (41.6)
Farming	No	1968 (84.9)
	Yes	351 (15.1)
Animal husbandry	No	1551 (66.9)
	Yes	768 (33.1)
Having a pet at home	No	1910 (82.4)
	Yes	409 (17.6)
Tick contact history	No	1949 (84)
	Yes	370 (16)
Duration of contact with tick contact (*n* = 370)	≤1 year	123 (33.2)
	2–4 years	114 (30.8)
	≥5 years	133 (35.9)
Hospitalization with suspected CCHF	No	2295 (99)
	Yes	24 (1)
Is there anyone in your family who was treated for CCHF?	No	2157 (93)
	Yes	162 (7)
If there is anyone in your family who was treated for CCHF, what is the health status? (*n* = 162)	Is alive	149 (92)
	Is dead	13 (8)
Is there anyone diagnosed with CCHF where you live?	No	1959 (84.5)
	Yes	360 (15.5)
If there is anyone diagnosed with CCHF where you live, what is the health status? (*n* = 360)	Is alive	242 (67.2)
	Is dead	118 (32.8)
Total		2319 (100.0)

**Table 3:** Comparison of anti-CCHF IgG seropositivity frequency in females and males by age groups of the participants.

Age groups	Female			Male			Total	
	*n*	Seropozitivity *n* (%)	*p* value^*∗*^	*n*	Seropozitivity *n* (%)	*p* value^*∗*^	*n*	Seropozitivity *n* (%)
20–29	169	3 (1.8)	0.001	124	2 (1.6)	<0.001	293	5 (1.7)
30–39	357	7 (2.0)		275	10 (3.6)		632	17 (2.7)
40–49	220	8 (3.6)		167	10 (6.0)		387	18 (4.7)
50–59	228	11 (4.8)		228	19 (8.3)		456	30 (6.6)
≥60 years	298	24 (8.1)		253	36 (14.2)		551	60 (10.9)
Total	1272	53 (4.2)		1047	77 (7.4)		2319	130 (5.6)

Anti-CCHFV IgG, anti-Crimean–Congo haemorrhagic fever virus immunoglobulin G. ^*∗*^Pearson chi-square tests.

**Table 4:** Logistic regression analysis of the descriptive characteristics of the participants in terms of anti-CCHFV IgG seropositivity (*n* = 2319).

Variables		Anti-CCHFV IgG		*p* value^*∗*^	COR (95% CI)	AOR (95% CI)
		Seropozitivity *n* (%)	Seronegativity *n* (%)			
Gender	Female	53 (4.2)	1219 (95.8)	0.001	Ref.	Ref.
	Male	77 (7.4)	970 (92.6)		1.83 (1.27–2.62)^*∗*^	2.53 (1.57–4.08)^*∗*^
Age groups	20–39 years	22 (2.4)	903 (97.6)	<0.001	Ref.	Ref.
	40–64 years	57 (5.6)	968 (94.4)		2.42 (1.47–3.99)^*∗*^	1.84 (1.05–3.20)^*∗*^
	≥65 years	51 (13.8)	318 (86.2)		6.58 (3.93–11.03)^*∗*^	4.05 (2.14–7.65)^*∗*^
Residental area	City center	43 (6)	671 (94)	0.561	Ref.	
	Districts	87 (5.4)	1518 (94.6)		0.89 (0.61–1.30)	
Education level	Illiterate-literate	46 (11.2)	366 (88.8)	<0.001	Ref.	Ref.
	Primary-secondary	71 (5.2)	1282 (94.8)		0.44 (0.30–0.65)^*∗*^	0.49 (0.30–0.80)^*∗*^
	High school-university	13 (2.3)	541 (97.7)		0.19 (0.10–0.36)^*∗*^	0.33 (0.14–0.76)^*∗*^
Marital status	Married	111 (5.5)	1901 (94.5)	0.014	Ref.	Ref.
	Single	3 (2.1)	139 (97.9)		0.37 (0.12–1.18)	0.89 (0.25–3.170)
	Divorced	16 (9.7)	149 (90.3)		1.84 (1.06–3.19)^*∗*^	1.30 (0.70–2.42)
Family income level	Bad	87 (7.3)	1102 (92.7)	<0.001	Ref.	Ref.
	Medium	33 (4.6)	685 (95.4)		0.61 (0.40–0.92)^*∗*^	0.93 (0.59–1.46)
	Good	10 (2.4)	402 (97.6)		0.32 (0.16–0.61)^*∗*^	0.71 (0.33–1.52)
Chronic disease	No	53 (4.9)	1034 (95.1)	0.151	Ref.	
	Yes	77 (6.3)	1155 (93.8)		1.30 (0.91–1.87)	
Smoking	No	86 (5.9)	1365 (94.1)	0.385	Ref.	
	Yes	44 (5.1)	824 (94.9)		0.85 (0.58–1.23)	
Alcohol use	No	120 (5.7)	1984 (94.3)	0.523	Ref.	
	Yes	10 (4.7)	205 (95.3)		0.81 (0.42–1.56)	
Obesity (BMI ≥ 30 kg/m^2^)	No	83 (5.9)	1333 (94.1)	0.503	Ref.	
	Yes	47 (5.2)	856 (94.8)		0.88 (0.61–1.27)	
Total		130 (5.6)	2189 (94.4)			

Anti-CCHFV IgG, anti-Crimean–Congo haemorrhagic fever virus immunoglobulin G; COR, crude odds ratio; AOR, adjusted odds ratio; CI, confidence interval; Ref, reference; ^*∗*^*p* value of Yates' corrected *X*^2^ test for anti-CCHFV IgG serology subgroup comparison.

**Table 5:** Logistic regression analysis of some CCHF-related risk factors for anti-CCHFV IgG seropositivity of the participants (*n* = 2319).

Variables		Anti-CCHFV IgG		*p* value^*∗*^	COR (95% CI)	AOR (95% CI)
		Seropozitivity *n* (%)	Seronegativity *n* (%)			
Location	Urban	70 (5.2)	1284 (94.8)	0.28	Ref.	
	Rural	60 (6.2)	905 (93.8)		1.22 (0.85–1.74)	

Farming	No	93 (4.7)	1875 (95.3)	<0.001	Ref.	Ref.
	Yes	37 (10.5)	314 (89.5)		2.38 (1.59–3.54) ^*∗*^	1.84 (1.18–2.86)^*∗*^

Animal husbandry	No	66 (4.3)	1485 (95.7)	<0.001	Ref.	Ref.
	Yes	64 (8.3)	704 (91.7)		2.05 (1.43–2.92)^*∗*^	1.64 (1.04–2.27)^*∗*^

Having a pet at home	No	104 (5.4)	1806 (94.6)	0.467	Ref.	
	Yes	26 (6.4)	383 (93.6)		1.18 (0.76–1.84)	

Tick contact history	No	93 (4.8)	1856 (95.2)	<0.001	Ref.	Ref.
	Yes	37 (10)	333 (90)		2.22 (1.49–3.30)^*∗*^	1.02 (1.03–2.29)^*∗*^

Duration of with tick contact	≤1 year	15 (12.2)	108 (87.8)	0.293	Ref.	
	2–4 years	13 (11.4)	101 (88.6)		0.93 (0.42–2.04)	
	≥5 years	9 (6.8)	124 (93.2)		0.52 (0.22–1.24)	

Hospitalization with suspected CCHF	No	121 (5.3)	2174 (94.7)	<0.001	Ref.	Ref.
	Yes	9 (37.5)	15 (62.5)		10.78 (4.62–5.13)^*∗*^	6.65 (2.70–16.43)^*∗*^

Is there anyone in your family who was treated for CCHF?	No	117 (5.4)	2040 (94.6)	0.165	Ref.	
	Yes	13 (8)	149 (92)		1.52 (0.84–2.77)	

If there is anyone in your family who was treated for CCHF, what is the health status? (*n* = 162)	Is alive	11 (7.4)	138 (92.6)	0.308	Ref.	
	Is dead	2 (15.4)	11 (84.6)		2.28 (0.45–11.61)	

Is there anyone diagnosed with CCHF where you live?	No	95 (4.8)	1864 (95.2)	<0.001	Ref.	Ref.
	Yes	35 (9.7)	325 (90.3)		2.11 (1.41–3.17)^*∗*^	1.66 (1.08–2.56)^*∗*^

If there is anyone diagnosed with CCHF where you live, what is the health status? (*n* = 360)	Is alive	22 (9.1)	220 (90.9)	0.563	Ref.	
	Is dead	13 (11)	105 (89)		1.24 (0.60–2.55)	

Anti-CCHFV IgG, anti-Crimean–Congo haemorrhagic fever virus immunoglobulin G; COR, crude odds ratio; AOR, adjusted odds ratio; CI, confidence interval; ^*∗*^*p* value of Yates' corrected *X*^2^ test for anti-CCHFV IgG serology subgroup comparison.

## Data Availability

The raw data used to support the ﬁndings of this study are available from the corresponding author upon request.
